# Variation of Ultimate Properties in Extruded iPP-Mesoporous Silica Nanocomposites by Effect of iPP Confinement within the Mesostructures

**DOI:** 10.3390/polym12010070

**Published:** 2020-01-02

**Authors:** Rosa Barranco-García, José M. Gómez-Elvira, Jorge A. Ressia, Lidia Quinzani, Enrique M. Vallés, Ernesto Pérez, María L. Cerrada

**Affiliations:** 1 Instituto de Ciencia y Tecnología de Polímeros (ICTP-CSIC), Juan de la Cierva 3, 28006 Madrid, Spain; rbarranco@ictp.csic.es (R.B.-G.); elvira@ictp.csic.es (J.M.G.-E.); ernestop@ictp.csic.es (E.P.); 2 PLAPIQUI (UNS-CONICET), Camino La Carrindanga km 7, Bahía Blanca 8000, Argentina; jressia@plapiqui.edu.ar (J.A.R.); lquinzani@plapiqui.edu.ar (L.Q.); valles@plapiqui.edu.ar (E.M.V.); 3 Comisión de Investigaciones Científicas de la Provincia de Buenos Aires (CIC), La Plata 1900, Argentina

**Keywords:** mesoporous silica, MCM-41 and SBA-15, iPP nanocomposites, thermal degradation, rheological properties, synchrotron SAXS measurements

## Abstract

Nanocomposites based on isotactic polypropylene (iPP) and mesoporous silica particles of either MCM-41 or SBA-15 were prepared by melt extrusion. The effect of the silica incorporated into an iPP matrix was firstly detected in the degradation behavior and in the rheological response of the resultant composites. Both were ascribed, in principle, to variations in the inclusion of iPP chains within these two mesostructures, with well different pore size. DSC experiments did not provide information on the existence of confinement in the iPP-MCM-41 materials, whereas a small endotherm, located at about 100 °C and attributed to the melting of confined crystallites, is clearly observed in the iPP-SBA-15 composites. Real-time variable-temperature Small Angle X-ray Scattering (SAXS) experiments with synchrotron radiation turned out to be crucial to finding the presence of iPP within MCM-41 pores. From these measurements, precise information was also deduced on the influence of the MCM-41 on iPP long spacing since overlapping does not occur between most probable iPP long spacing peak with the characteristic diffractions from the MCM-41 hexagonal nanostructure in comparison with existing superposition in SBA-15-based materials.

## 1. Introduction

Behavior of liquids and solids in very small pores has been a relevant topic, from both a fundamental and practical perspective, for decades [1]. Phenomena, such as the glass transition, phase separation crystallization, and the subsequent melting under confinement, were investigated in order to learn the effect of finite size constraints on bulk properties. This interest also spread to include other materials, such as polymers, with the arrival of nanotechnology in order to attain and understand new physical properties on the molecular scale. The development of polymer-based nanocomposites, thin films and coatings, nanolithography in semiconductor manufacturing, etc. promoted the basic knowledge of the involved molecular phenomena, which are considered critical to the success of the confinement understanding.

Isotactic polypropylene (iPP) is used in a wide variety of applications, such as in the automotive and aerospace industries, because it shows a much desirable versatility and useful physical properties, such as stiffness and strength. Incorporation of specific fillers, leading to the obtainment of micro or nanocomposites, can contribute to the enhancement of some of those excellent properties and to allow spreading out even more its applicability.

Confinement of iPP chains within the ordered spaces present in SBA-15 mesoporous silica was proved recently by Small-angle X-ray scattering (SAXS) using synchrotron radiation, these measurements constituting a reliable and powerful tool [2]. The iPP macrochains filled out those nanometric channels when composites were obtained not only by in situ polymerization [3] but also, unexpectedly, by extrusion [2,4] of the two components, the molten iPP and the SBA-15 particles. Confinement was deduced through the observation of a noticeable discontinuity in the intensity of the first order (100) diffraction of the SBA-15 related to its characteristic hexagonal arrangement. The upward step took place in the temperature interval ranging from 95 to 120 °C, which was fully in agreement with the one noticed for a small endotherm exhibited in the DSC experiments [2,3,4]. Earlier investigations on several SBA-15 composites [5,6,7] described that the intensity of the SBA-15 diffraction was dependent on the eventual scattering contrast between walls and inside of the mesopores. These variations were ascribed to those changes in the electron density from iPP chains arranged inside the SBA-15 particles, which were semicrystalline at low temperatures or fully amorphous after the melting of these iPP crystallites existing within mesoporous channels. Moreover, the intensity of that first order reflection was strongly dependent on SBA-15 content [2,4]. These results confirmed the ones described in previous works performed at room temperature on in situ polymerized nanocomposites of poly(*N*-isopropylacrylamide)/SBA-15 [8] as well as on polyethylenimine (PEI)-based composites wet impregnated with either MCM-41 or SBA-15 [7,9].

Regarding the existence of a small endothermic event in DSC, it should be said that this process was also observed for in situ polymerized materials based on ultra-high molecular weight polyethylene (UHMWPE) and SBA-15 [10,11] as well as in nanocomposites based on high density polyethylene (HDPE) and MCM-41. The temperature range was shifted to lower values [12,13,14] in the latest ones. These secondary processes were associated with the melting of crystallites with significantly smaller size than the thicker ones that melt at around 130 °C during the main endotherm [1]. These observations seemed to point out that pore size in the silica plays a key role since crystallites cannot grow more than the nanometric spaces where chains are confined.

There are not many articles dealing with metallocenic iPP and mesoporous silicas and even less analyzing confinement effects. Most of them are related to the in situ polymerization topic. An approach implied the pretreatment of zirconocene with methylaluminoxane (MAO) before impregnating the catalyst [15]. Other studies showed that the amount of catalyst that can be immobilized increased by pretreating the support with MAO [16] and a superior catalytic activity was observed during polymerization. Improvements in this parameter were also achieved by substituting the microsized MCM-41 by its nanoparticles [17]. Nanocomposites of PP and MCM-41 nanoparticles achieved by in situ polymerization were described once in the literature [18] but without evaluation of properties of the resulting materials. Thus, the objective of this research is to obtain a deeper understanding on the influence of the incorporation by extrusion of either MCM-41 or SBA-15 particles to the iPP and the evaluation in the attained (nano)composites of the differences in terms of thermal stability, rheological behavior, crystalline characteristics, and confinement effects. Several techniques were used in this research, including size exclusion chromatography (SEC), scanning electron microscopy (SEM), wide and small angle X-ray Scattering (WAXD and SAXS, respectively) with synchrotron radiation, differential scanning calorimetry (DSC), thermogravimetry (TGA), and rheological experiments in the molten state.

Could polypropylene chains go inside the smaller nanometric spaces of MCM-41 helped by the shear forces applied during the extrusion process? A response to this question will contribute to the understanding of how iPP confinement affects its structure and dynamics and also its thermal degradation.

## 2. Materials and Methods

### 2.1. Materials and Chemicals

A commercially available metallocene-catalyzed isotactic polypropylene (Metocene HM562P: melt flow index of 15 g/10 min at 230 °C/2.16 kg, kindly supplied by LyondellBasell) was used in the present research as polymeric matrix. The MCM-41 and SBA-15 particles were purchased from Sigma-Aldrich (specific surface area, *S*_BET_^MCM−41^ = 966 m^2^/g and *S*_BET_^SBA−15^ = 619 m^2^/g; average mesopore diameter [19], *D_p_*^MCM−41^ = 2.9 nm and *D_p_*^SBA−15^ = 8.0 nm) and were used as received.

### 2.2. (Nano)composite and Film Preparation

Composites with different contents in particles of MCM-41 (0%, 2%, 4%, 8%, and 14% in weight) or of SBA-15 (0%, 1%, 4%, 8%, and 13% in weight) and iPP were processed by melt extrusion in a corotating twin-screw microextruder (Rondol). They were named as iPPMCM2, iPPMCM4, iPPMCM8 and iPPMCM14 for the materials prepared with MCM-41 and iPPSBA1, iPPSBA4, iPPSBA8 and iPPSBA13 for the composites obtained with SBA-15. The extruded homopolymer was labeled as iPP. Both iPP polymer and mesoporous silica (MCM-41 or SBA-15) were dried previously for 24 h under vacuum at 110 °C. A screw temperature profile of 115, 170, 180, 185 and 190 °C was used from the hopper to the die, being the length-to-diameter ratio 20:1. Then, films were obtained by compression molding at 190 °C and at 30 bar for 6 min in a hot-plate Collin (model 200 × 200) press. A relatively fast cooling process (rate around 80 °C/min) was applied between plates under pressure (30 bar) to the different films from their melt to room temperature.

### 2.3. Sample Characterization and Properties

The molecular weight and molecular weight distribution for the isotactic polypropylene used as polymeric matrix in this investigation were obtained by Size Exclusion Chromatography (SEC) using a Waters 150-C ALP/GPC equipped with a set of three PL-GEL MIXED-A columns from Polymer Labs. The solvent used was 1,2,4-trichlorobenzene (TCB) at 135 °C with 1 mL/min flow. The apparent molecular weight of the polymer was estimated following the standard calibration procedure using monodisperse polystyrene samples and the corresponding Mark-Howink coefficients for polypropylene [20]. The values obtained after its extrusion were 173,000 g/mol and 2.0 for polydispersity.

Experiments of high-resolution field emission scanning electron microscopy (FESEM) were carried out in a S-8000 Hitachi equipment at room temperature in different cryo-fractured sections of composites at distinct mesoporous content. Those thin sections of around 40 nm were cut by cryo-ultramicrotomy (Leica EM UC6) at −120 °C and deposited in a holder.

Thermogravimetric analysis (TGA) was performed in a Q500 equipment of TA Instruments under air or nitrogen atmosphere at a heating rate of 10 °C/min. Determination of the silica amount (MCM-41 or SBA-15) in these nanocomposites prepared by extrusion was carried out as an average of the values obtained under the two atmospheres. The resulting values of the silica content are listed in Table 1. Calorimetric analyses were carried out in a TA Instruments Q100 calorimeter connected to a cooling system and calibrated with different standards. The sample weights were around 3 mg. A temperature interval from −40 to 180 °C was studied under an inert atmosphere of nitrogen at a heating rate of 20 °C/min. For the determination of the crystallinity, a value of 160 J/g was used as the enthalpy of fusion of a perfectly crystalline material [21,22,23].

Real-time variable-temperature simultaneous SAXS/WAXD experiments were carried out with synchrotron radiation in beamline BL11-NCD at ALBA (Cerdanyola del Vallès, Barcelona, Spain) at a fixed wavelength of 0.1 nm. An ADSC detector was used for SAXS (off beam, at a distance of 294 cm from sample) and a Rayonix one for WAXD (at about 19 cm from sample, and a tilt angle of around 30 degrees). A Linkam Unit, connected to a cooling system of liquid nitrogen, was employed for the temperature control. The calibration of spacings was obtained by means of silver behenate and Cr_2_O_3_ standards. The initial 2D X-ray images were converted into 1D diffractograms, as function of the inverse scattering vector, *s* = 1/*d* = 2 sin *θ*/λ. Film samples of around 5 × 5 × 0.1 mm were used in the synchrotron analysis.

Rheological characterization was carried out in small-amplitude oscillatory shear mode using a dynamic rotational rheometer TA Instruments ARG2 (New Castle, DE, USA). The tests were performed under nitrogen atmosphere using parallel plates of 25 mm in diameter, at a frequency range between 0.1 and 100 rad/s, and a temperature interval of 180–220 °C. All tests were carried out at small stresses in order to assure the linearity of the dynamic responses [24]. These stresses were selected performing initial stress dynamic sweeps at constant frequency of 1 rad/s.

## 3. Results and Discussion

**Figure 1:**
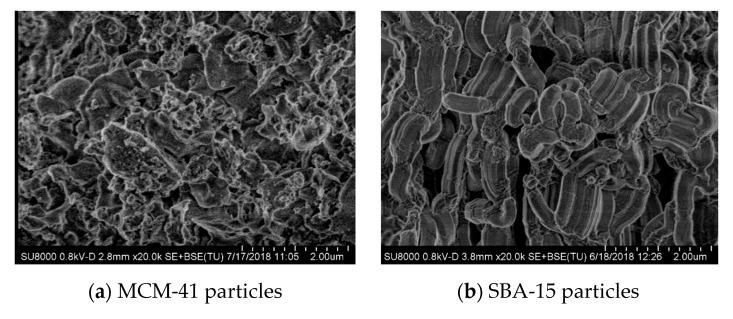

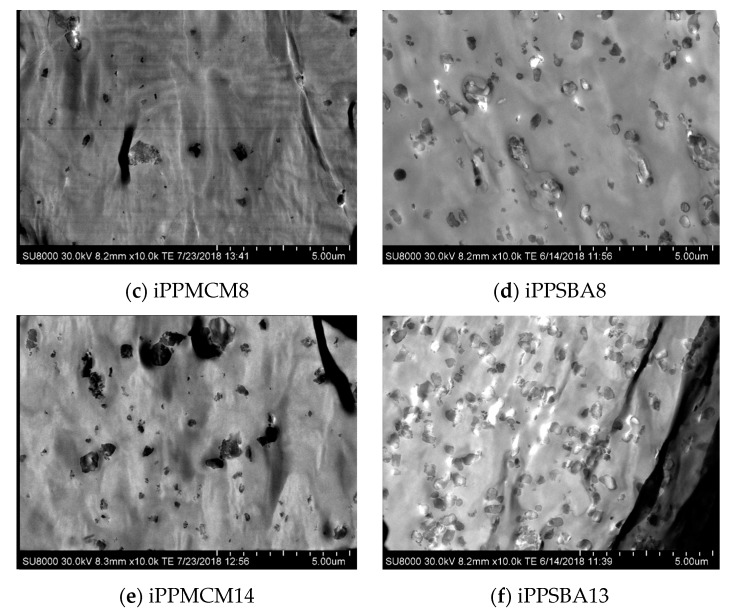
FESEM micrographs for different materials. On the top: pristine MCM-41 (**a**) and SBA-15 (**b**) particles; in the middle: iPPMCM8 (**c**) and iPPSBA8 (**d**) composites; and, on the bottom: iPPMCM14 (**e**) and iPPSBA13 (**f**).

Figure 1 shows the FESEM micrographs for the pristine particles of MCM-41 and SBA-15 (pictures at the top) as well as for several of the composites prepared by extrusion based on iPP and both mesoporous silicas (middle and lower pictures). Important differences are observed between the two neat silicas. Particles of MCM-41 exhibit their common irregular shape [19,25] while the ones for SBA-15 show a vermicular elongated contour [10,19] with an average size of 350 nm wide and 0.9 μm long.

Some variations are also seen when composites at almost same load are compared. A rather homogeneous dispersion of SBA-15 particles within the materials is clearly noted together with the absence of agglomerates with large size in the iPPSBA8 and iPPSBA13 composites. Moreover, an obvious increase in the number of SBA-15 particles is noticed as its content is raised in the final hybrid (precise compositions determined by TGA measurements are detailed in Table 1). Dispersion seems to be; however, less uniform in the materials containing MCM-41 microsized particles, independently of the content. In addition, MCM-41 aggregation is detected in these materials in an extent larger than in the ones prepared by using SBA-15. Despite these differences, the particle distribution and size of aggregates for both mesoporous silicas are sufficiently suitable in the final materials, mainly taking into account that they are incorporated into a non-polar polymeric matrix like the iPP, using melt extrusion as processing approach without aid of a compatibilizer agent. Existence of aggregates was also described even in composites prepared by in situ polymerization based on non-polar polyethylene and pristine microsized MCM-41 [25] or SBA-15 particles [10].

**Figure 2:**
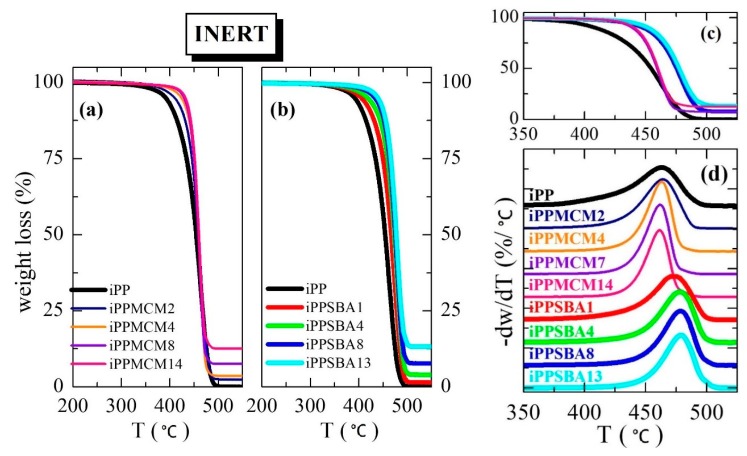
TGA curves under inert atmosphere for the materials prepared from iPP with particles of MCM-41 or SBA-15 ((**a**,**b**) plots, respectively) with different silica contents. Plot (**c**) shows a comparison between several samples with similar mesoporous amounts (the color code is maintained as for plots (**a**,**b**)); and plot (**d**) depicts TGA derivatives under this inert atmosphere.

**Figure 3:**
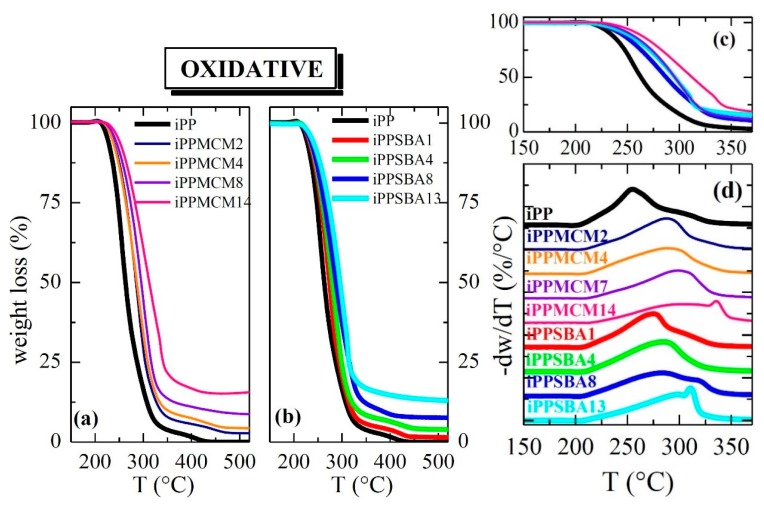
TGA curves under oxidant atmosphere for the materials prepared from iPP with particles of MCM-41 or SBA-15 ((**a**,**b**) plots, respectively) with different silica contents. Plot (**c**) shows a comparison between several samples with similar mesoporous amounts (the color code is maintained as for plots (**a**,**b**)); and plot (**d**) depicts TGA derivatives under this oxidative atmosphere.

Figure 2 and Figure 3 show the TGA curves under inert and oxidative atmosphere, respectively, for the materials prepared from iPP with either MCM-41 (plots (a)) or SBA-15 (plots (b)) particles with different silica contents. The final content in mesoporous silica, listed in Table 1, was determined from these experiments as average value of those deduced from the tests performed under these two different environments.

Figure 2 and Figure 3 also display the degradation behavior in a broad temperature range that allows realizing the effect of the two silicas in the iPP decomposition process of these materials. First of all, incorporation by extrusion of mesoporous silica particles into iPP increases its thermal stability independently of the experimental atmosphere (Figure 2 under inert and Figure 3 under oxidant conditions, respectively) and type of silica used. Nevertheless, presence of MCM-41 or SBA-15 leads to opposite trends in the iPP degradation depending on the surrounding ambient.

Data in Table 1 together with comparison for the materials with the highest silica incorporations, depicted in the plot (c) of Figure 2, clearly show that MCM-41 stabilizes the iPP decomposition in less extent than SBA-15 particles under inert conditions. In fact, the maximum degradation temperature in these iPP−MCM-41 materials is similar or even slightly inferior to that observed in the neat iPP, as displayed in plot (d) of Figure 2. Differences are found at the beginning of the process, being these quantified in Table 1 as temperature for a mass loss of 10% by weight (T10%). Accordingly, this T10% is noticeably moved to superior temperatures in the iPP−SBA-15 composites, exhibiting values of T10% and T^max^ higher than iPP and iPP−MCM-41 hybrids, as noticed in plot (d) of Figure 2 and in Table 1. Under these conditions, all of the specimens display a single main stage of decomposition in the temperature range from 300 to 550 °C, and improvement in the iPP thermal performance associated with presence of mesoporous particles is dependent on their content. The iPP degradation mechanism was reported [26] not to change because of the SBA-15 particles although an effective delay in the build-up of the distinctive species was observed.

TGA curves of these composites under air exhibit, at least, two degradation processes in the temperature interval ranging from 200 to 375 °C, as depicted in Figure 3 (plots (a), (b) and (d)). As already described [27,28], the preliminary reaction in polyolefins during thermal oxidation is the alkyl radicals formation from polymeric chains followed by the reaction of those alkyl radicals with oxygen to form hydroperoxides, which can decompose to alkoxyl radicals. Then, the alkoxyl radicals abstract hydrogen from the chain and other alkyl radical forms. Finally, various carbonyl species are generated.

Figure 3 shows that degradation is dependent on either silica content or its pore size. The former shifts its location to higher temperatures while the latest provokes that decomposition takes place at lower temperature for materials containing SBA-15, which is the silica with significantly larger pore diameter. Consequently, a considerable thermal stabilization for the iPP matrix is achieved under oxidative conditions if MCM-41 particles are added instead of SBA-15 silica, as seen from the different plots of Figure 3 together with data listed in Table 1.

**Figure 4:**
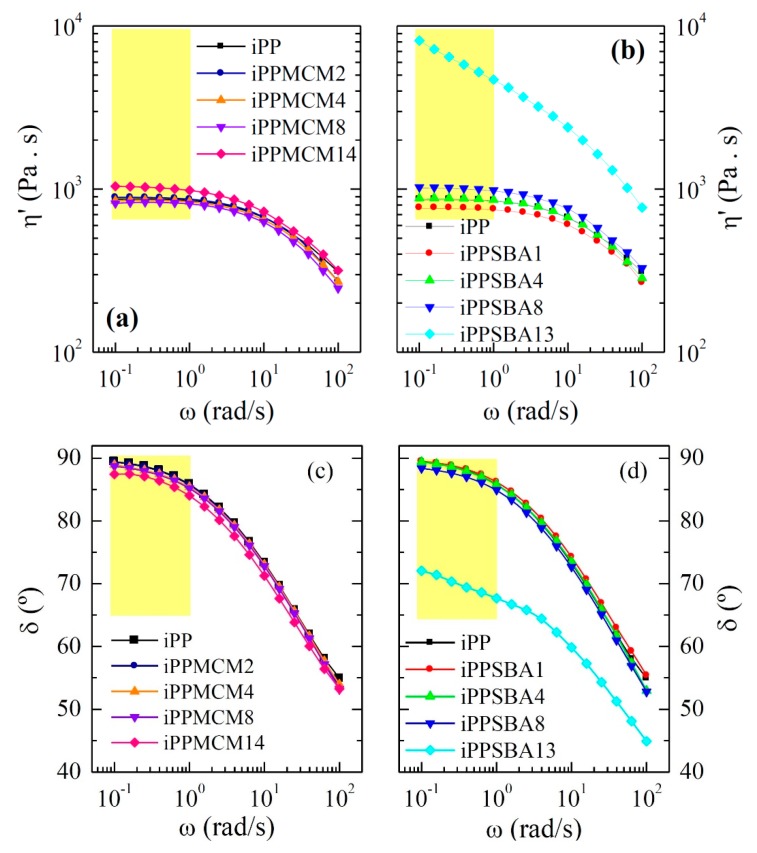
Reduced frequency dependence at 200 °C of viscosity, η′, (**a**,**b**) plots, and phase δ angle, (**c**,**d**) plots, for iPP–MCM-41 and iPP–SBA-15 composites at different silica contents.

The positive impact in the iPP thermal stability in iPP−SBA-15 composites was assumed [26] to be related to an increase of the molten state viscosity in the materials by incorporation of silica. This rise was little for SBA-15 contents up to 8 wt.%, being more significant for higher SBA-15 content. Presence of SBA-15 and existence of iPP chains within their channels leaded to a hindrance of air diffusion into the bulk and, thus, to a postponement in the oxidation of iPP chains. In addition, high contents in SBA-15 provoked air diffusion through distorted pathways molten PP matrix. That assumption about the melt viscosity differences was checked by studying the rheological behavior of the different composites. Figure 4 shows the effect of both mesoporous silicas on dynamic viscosity (η′) and phase angle (δ).

The iPPSBA13 material, containing the highest amount in SBA-15, presents a very considerable increase in viscosity in the whole frequency range. On the contrary, viscosity remains almost constant in the iPP−MCM-41 composites, except for the iPPMCM14 where η′ is slightly raised. This different behavior could be associated with the distinct pore diameter existing within particles from these two mesoporous silicas, since both display parallel one-dimensional channels that are disposed in ordered hexagonal arrangements [29,30]. Presence of larger or smaller pores along these mesoporous particles might involve important changes, mainly related to the capability of iPP chains to be included within those bare tubes by the shear forces applied during processing. By means of evolved gas analysis, pore filling was described to be different between MCM-41 and SBA-15 in materials based on mesoporous silicas with hydroxyl-functionalized polypropylene [31], being much smaller in the former. The existence of more iPP chains within SBA-15 particles [2,3,4] could lead to that important variation in those two rheological parameters. These pristine iPP macromolecules coming out from the mesoporous SBA-15 could promote the matrix−filler interactions and boost an improvement in the interfacial matrix−filler adhesion, both being responsible for the rise in viscosity and δ reduction. Truthfulness of this assumption should also involve changes of dependence of storage and loss moduli on frequency.

**Figure 5:**
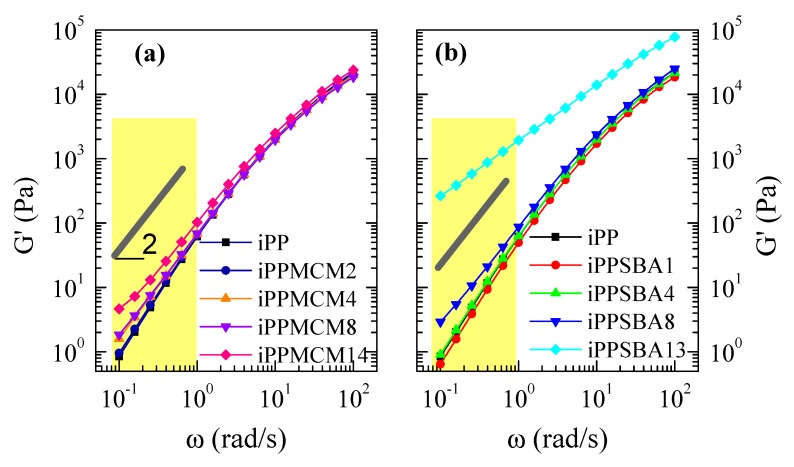
Reduced frequency dependence at 200 °C of storage, G’(ω), modulus for iPP–MCM-41 and iPP–SBA-15 composites at different silica contents, (**a**) and (**b**) plots, respectively. For guidance, straight lines of slope = 2 were also added.

Figure 5 represents the variation on frequency of elastic (G′) component of shear modulus |G*| for the different materials under study. G″ values (not shown) are higher than G′ ones in the whole frequency range. This means that terminal flow region is present in all the samples. A slight increase of both magnitudes is observed as growing amounts of mesoporous silica are loaded in the final material, either for iPP−MCM-41 or iPP−SBA-15 composites. This characteristic is more evident for the highest silica contents and in the iPP−SBA-15 hybrids. In fact, the behavior exhibited by iPPSBA13 is significantly different. Contrary to the cases of iPP homopolymer and the rest of iPP−MCM-41 or iPP−SBA-15-based materials, iPPSBA13 does not show a thermo-rheological simple response. Accordingly, the G′ and G″ isotherms do not accomplish time-temperature superposition principle in this frequency range and the corresponding master curve cannot be built. A deviation is exhibited [24] from the common dependence of G′ and G″ power-laws, ω^2^ and ω respectively, as seen for G′ in Figure 5.

This deviation is also clearly observed in iPPMCM14 and iPPSBA8, although both do behave as thermo-rheologically simple materials. Thus, a diminishment in slopes is attained and G′ and G″ values come closer at the lowest frequencies. These features point out the beginning of a transition from a liquid to a solid-like behavior. This particular rheological behavior is attributed to the development of a momentary network ascribed to percolation of fractal filler aggregates joined together with bridging polymeric macrochains [32,33]. It is designated as rheological percolation. The existence of percolation was also described in other iPP-based composites [34] although distinct trends were also found in the literature [35]. These differences could be associated with particle shape, size, state of dispersion and concentration. Each material shows its own characteristics and there are not previous articles related to iPP−mesoporous silica hybrids. Filler aspect ratio is known to be an important variable and percolation threshold is reduced with increasing ratios.

All these rheological results seem to indicate that inclusion during extrusion of iPP is into the mesoporous MCM-41 more difficult than within the SBA-15 particles, because the pore diameter is considerably smaller in the former. This difficulty would be also responsible for the analogous results found between iPPMCM14 and iPPSBA8 in spite the former contains almost double amount of silica. The fact that MCM-41 pores may be emptier than those in SBA-15 particles could also justify the aforementioned effect of MCM-41 on the oxidative iPP degradation, since those voids would favor air capture, their interaction with hydroxyl groups from silica during decomposition and the resultant reduction of air amount in the medium. This disturbance in air diffusion would contribute to postpone oxidation of iPP macrochains and to shift degradation to higher temperatures in the iPP−MCM-41 materials. However, are MCM-41 pores filled with the iPP chains or not? An absolute response cannot be provided by dependence on frequency of the rheological magnitudes in the iPP−MCM-41 composites, since they change only slightly with silica content. This fact seems to indicate that there is not a significant amount of iPP chains filling the MCM-41 channels in contrast to that observed in the iPP−SBA-15 materials.

**Figure 6:**
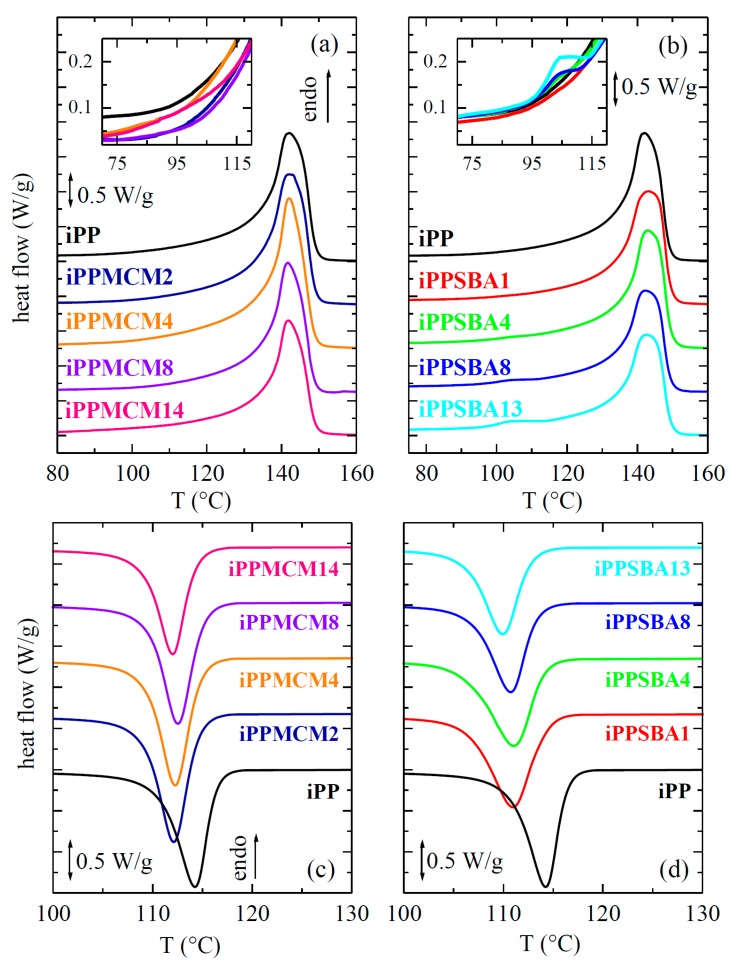
DSC endotherms related to the first melting run, (**a**,**b**) plots, shifted along Y axis for a better visualization, for samples prepared from iPP with MCM-41 and SBA-15 particles, respectively. DSC exotherms attained during crystallization process, (**c**,**d**) representations, for the materials extruded prepared from iPP with MCM-41 and SBA-15 silica, respectively.

As mentioned in the introduction, DSC measurements were previously used as an approach of easy availability for knowing the existence of polymeric chains within particles from either MCM-41 or SBA-15. The DSC curves for the present composites are displayed in Figure 6. The upper plots (a) and (b) show the first melting curves for all of specimens. They exhibit a main endotherm at about 142 °C (see results in Table 2). This process is associated with the melting of the monoclinic crystallites (as will be commented below), which are the ones commonly developed under the fast cooling conditions applied during the film processing. Total enthalpy involved in this primary process seems to remain rather unchanged with type and content of silica used in the final material. Crystallinity estimation was performed after normalization of heat flow to the actual amount of polypropylene at each specimen. Values obtained are quite similar for the distinct composites.

Another small endothermic process, at around 100 °C, is observed in the iPP−SBA-15 materials additionally to that intense melting. It was related to those small crystals that are able to be developed within the nanometric SBA-15 channels. They melt at low temperature because of their significant smaller size. On the contrary, this little endothermic event is not seen in the iPP−MCM-41 composites (the appearance of that endotherm is expected in the iPP−MCM-41 specimens at lower temperature than in those hybrids with SBA-15 particles, similarly to the features described for polyethylene materials with either MCM-41 [12,13,14] or SBA-15 [10,11]). Thus, it seems that there are not iPP chains within MCM-41 channels in a significant amount. Variation of rheological parameters with frequency showed, however, some slight changes with MCM-41 content. Consequently, the absolute statement of absence of iPP chains filling these nanometric MCM-41 pores cannot be established from DSC. Then, SAXS experiments turn out mandatory since they were proved as a powerful and reliable tool for the confinement analysis in iPP-based materials [2,3,4]. Their results will be discussed below in detail.

Lower plots, (c) and (d), in Figure 6 display the crystallization process during the subsequent cooling from the melt. Similar trend is observed in all cases, basically independent of the type and content in mesoporous silica. Accordingly, iPP crystallization is postponed in all the composites and takes place at lower temperatures. MCM-41 particles seem to delay the iPP ordering process in a smaller extent and, thus, *T_c_* values, reported in Table 2 for the different contents, are slightly higher than those exhibited by the materials incorporating SBA-15 silica. The opposite crystallization behavior was described in HDPE/MCM-41 nanocomposites prepared by in situ polymerization using either pristine or modified with silanes MCM-41 particles. Thus, a nucleating effect was observed, which was minimized with decoration. The shift of *T_c_* to higher temperatures was also found at the same silica range in iPP nanocomposites with SBA-15 synthesized by in situ polymerization [3]. In these latest materials, a crystallization hindrance was seen only for SBA-15 amounts greater than 20 wt.%. It should be commented that molecular weight of that synthesized iPP was considerably inferior [3] to the one of this commercial iPP here used.

Do MCM-41 and SBA-15 particles trigger the same iPP crystalline lattice in the resultant composites? An interesting polymorphic behavior is well known to take place in iPP derivatives. Thus, the iPP can crystallize into different cells by changing microstructural characteristics, crystallization parameters and other factors, such as the incorporation of specific nucleants [37,38,39,40]. Three different polymorphic modifications, α, β and γ, were described together with a phase of intermediate or mesomorphic order obtained by fast quenching [37,38,39,40,41,42,43]. In addition to these four modifications, a trigonal form was firstly reported in the literature [44] in 2005 for isotactic copolymers of propylene with high contents of 1-hexene [45,46] or 1-pentene [47,48], in propylene terpolymers with 1-pentene and 1-hexene [49,50] as comonomers, and in propylene terpolymers with 1-pentene and 1-heptene [51], all synthesized by using metallocene catalysts.

The iPP−SBA-15 composites prepared by extrusion showed the coexistence of orthorhombic and monoclinic modifications in specimens slowly crystallized [2] while they only exhibited the monoclinic polymorph in those fast cooled from the melt, independently of they were extruded or prepared by means of in situ polymerization [3,4]. The driving force pushing the development of those two or one crystalline lattices, respectively, was crystallization rate, rather than the presence of that mesoporous silica.

**Figure 7:**
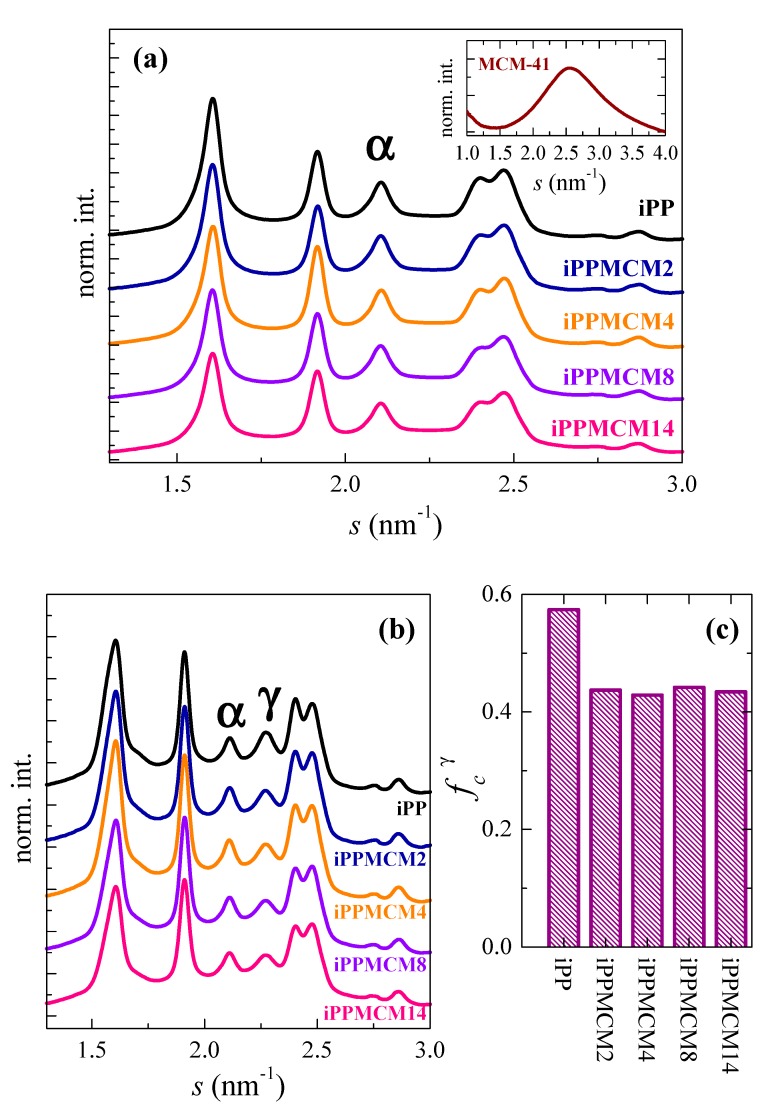
(**a**) Synchrotron WAXD 1D diffractograms of initial iPP−MCM-41 materials and the MCM-41 silica (inset); (**b**) WAXD 1D profiles at room temperature after crystallization at 20 °C/min from the melt; (**c**) orthorhombic γ content estimated from the WAXD 1D profiles at room temperature after crystallization at 20 °C/min.

Figure 7a shows the WAXD patterns at room temperature of the different iPP−MCM-41 samples. MCM-41 silica, represented in the inset, is completely amorphous and its halo overlaps with the iPP patterns of the homopolymer and the different composites in this scattering range. No significant differences are observed between the neat iPP and the specimens containing MCM-41. All of them crystallize exclusively into monoclinic crystals, showing their characteristic diffractions. Situation changes considerably after their melting and subsequent crystallization at 20 °C/min, which is a rate much lower than that imposed during the fast cooling applied in the processing of films (approximately 80 °C/min). Now, coexistence of monoclinic and orthorhombic polymorphs is clearly seen in Figure 7b as consequence of reduction in the rate, as deduced from observation of the characteristic (130)^α^ and (117)^γ^ reflections at *s* around 2.115 and 2.275 nm^−1^, respectively.

Development of the γ modification is boosted in the metallocene iPPs [52,53] if its crystallization takes place slowly. Figure 7c represents the orthorhombic content deduced from those profiles represented in Figure 7b. Presence of MCM-41 within the iPP matrix hinders significantly the formation of this polymorph. In fact, its amount is reduced from 57% of the total crystals in the neat iPP to around 43% for the MCM-15 composites, being the percentage quite similar for all composites. This feature is somehow different to the results found in slowly crystallized iPP−SBA-15 composites, where the presence of SBA-15 silica reduced only very slightly the amount in orthorhombic crystals [2]. It is true that crystallization rate in those iPP−SBA-15 samples was much smaller than that applied for these iPP−MCM-41 composites.

Determination of overall crystallinity together with that for the individual content in each polymorph requires different subtractions: firstly, of the amorphous MCM-41 contribution, followed next of that coming from the amorphous iPP halo [23,43]. The values achieved after normalization to the actual iPP content in the composites are rather similar for all them, as listed in Table 2. They turn out also analogous to the crystallinity degrees estimated for the SBA-15 composites.

Another aspect to solve is to learn, without a doubt, whether or not there are iPP chains within the MCM-41 mesostructure. On one hand, the small endotherm usually observed in PE−MCM-41 [12,13,14], UHMWPE−SBA-15 [10,11] and iPP−SBA-15 nanocomposites [2,3,4], related to the melting of polymeric chains inside the pores, does not appear in the DSC curves during the first melting process of these iPP−MCM-41 materials. On the other hand, dependences on frequency of their rheological parameters seem to indicate the beginning of a transition from a liquid to a solid-like behavior, which could be ascribed to presence of iPP chains within MCM-41 channels. It must be considered that although some iPP macrochains can fill out these MCM-41 nanometric pores, the amount must be much lower than that present in the SBA-15 channels because of the significantly smaller diameter in the former. Moreover, extrusion was used for preparation of these iPP−MCM-41 composites, which can be an unfavorable approach for the inclusion of iPP within the nanometric pores compared with the in situ polymerization. As mentioned in the introduction, real-time variable-temperature SAXS measurements using synchrotron radiation were a very useful and conclusive tool to learn on the presence of iPP chains within the SBA-15 mesostructure [2,3,4]. Accordingly, results from these experiments are now discussed for the iPP−MCM-41 composites.

**Figure 8:**
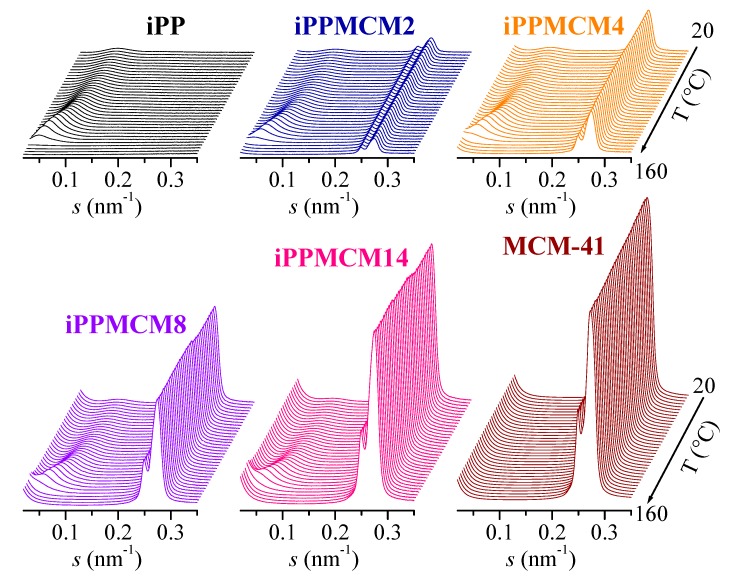
Lorentz-corrected synchrotron SAXS 1D diffractograms for the melting at 20 °C/min of the indicated samples. The vertical scale for neat MCM-41 was divided by a factor of 7. For clarity, only one every two frames is plotted.

Figure 8 shows the Lorentz-corrected synchrotron SAXS 1D profiles attained from 20 to 160 °C during the initial melting at 20 °C/min in the different samples of interest. First of all, it should be commented that the vertical scale was divided for the pristine MCM-41 by a factor of 7 in order to focus the attention in the materials where silica is the minor component. Secondly, the high intensity and collimation of synchrotron radiation used proves that the first (100) order, corresponding to MCM-41 characteristic hexagonal arrangement, which is commonly asymmetric, is really split into two contributions, appearing at 0.249 nm^−1^ and 0.274 nm^−1^, for this commercial MCM-41 silica. Superior orders (not shown) display the same feature.

Profiles for the different composites exhibit clearly two distinct regions: the one observed at the lowest s values (at around 0.085 nm^−1^ at 20 °C) and the zone located in the surrounding of 0.25 nm^−1^, perceptible as double peak. The former is associated with the long spacing (*L*) ascribed to lamellar iPP crystals while the latest arises from the above commented hexagonal arrangement for the MCM-41 particles. At a first approximation, all the samples at the low s interval, except obviously the neat MCM-41 silica, show at room temperature a broad *L* peak of small intensity, which location is moved to smaller s values and becomes considerably more intense as increasing temperatures. No important changes seem to take place with the MCM-41 content. Regarding the double peak ascribed to the MCM-41 first order, its intensity is, obviously, significantly dependent on the amount of mesoporous silica, as noticed clearly in Figure 8.

**Figure 9:**
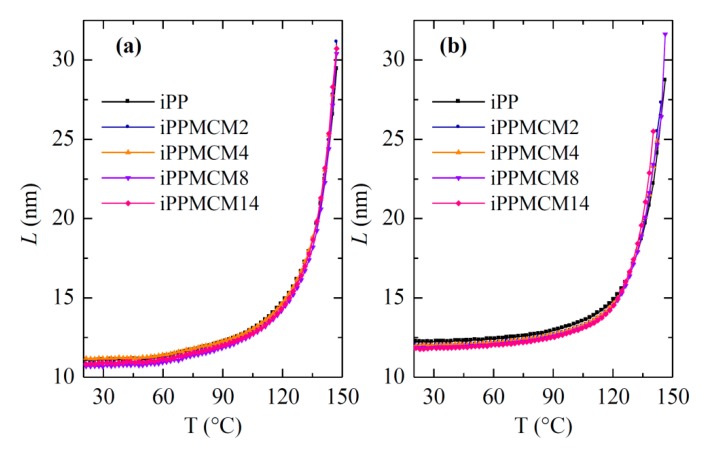
Dependence of Lorentz-corrected long spacing on temperature for the different iPP−MCM-41 specimens during (**a**) first melting and (**b**) melting after crystallization at 20 °C/min.

A detailed assessment of the long spacing for the iPP component confirms a quite small effect of presence and content of MCM-41 on their values, as seen in Figure 9. The first melting of the initial samples proves that the *L* values range from 10.7 to 11.1 nm at room temperature (crystal size, *l_c_*, between 6.4 and 6.7 nm, assuming a simple two phase model [36], as seen in Table 2). These are similar to those reported for the iPP−SBA-15 composites [4] with an analogous thermal treatment. Moreover, two regions are noted depending on temperature in all samples: an initial one, up to around 120 °C, with a moderate increase of *L*; and a final one, with a very important rise of *L* values, which is attributed to the crystal thickening phenomenon. This last stage coincides with the main melting endotherm (see Figure 6a) and it is ascribed to the usual melting-recrystallization processes.

Crystallization at 20 °C/min leads to slightly thicker crystals, whose sizes at room temperature range from 11.7 to 12.1 nm, as seen in Figure 9b. It should be reminded that some of these crystallites are orthorhombic, as deduced from Figure 7b,c. These *L* values show a slight decrease as increasing MCM-41 content. Their dependence on temperature again displays two distinct regions, as aforementioned for the first melting. Now, melting-recrystallization processes occur in a less extent since crystallization rate was considerably smaller than that applied during processing of the films, so that now the original crystals are more perfect.

**Figure 10:**
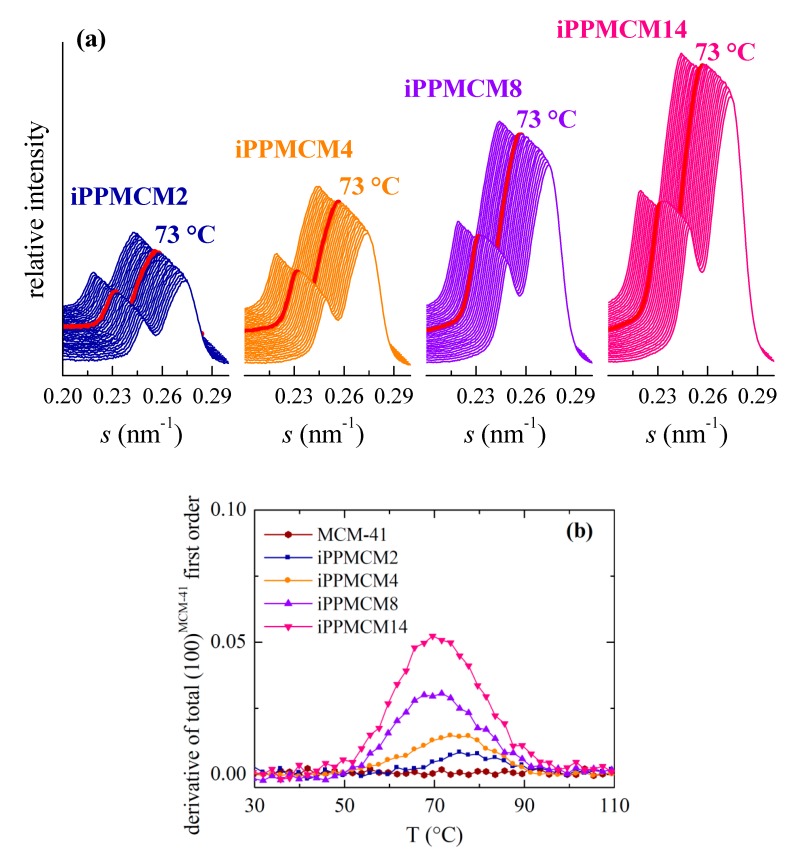
(**a**) SAXS profiles during the first melting process from 20 (rear profiles) to 160 °C (front patterns) for the distinct iPP−MCM-41composites at the s interval corresponding to the first order of MCM-41 hexagonal arrangement. Curves were shifted for a better understanding. (**b**) Dependence with temperature of derivative of total area from SAXS peaks for the first order in the different iPP nanocomposites and neat MCM-41 during the melting experiments.

The thorough examination of the s interval at higher values shows a rather systematic behavior of the SAXS peak corresponding to the MCM-41 first order of its hexagonal arrangement in the different iPP−MCM-41 composites, which displays two well differentiated contributions, as mentioned above. Thus, although the position of these two peaks remains practically unchanged with temperature, their intensities, however, undergo a significant and regular increase for all the iPP nanocomposites in the temperature range from around 55 to 90 °C, as clearly observed in Figure 10a,b. This latest plot represents the derivative of the total area of those two peaks corresponding to the first order of that hexagonal arrangement shown by this commercial MCM-41. Interestingly, that increase is not observed for the neat MCM-41 silica (as also depicted in Figure 8), which exhibits a location, width, and intensity practically constant in the temperature interval of interest.

Appearance of a discontinuity upon temperature was already described in iPP-based materials incorporating SBA-15 prepared both by in situ polymerization and by extrusion [2,3,4]. It was assumed that the first order of the hexagonal SBA-15 morphology was able of detecting the difference in scattering contrast between the walls and the inside of the SBA-15 channels, and this latest one is dependent on the semicrystalline or completely amorphous state of the iPP macrochains in the interior of pores due to differences in electronic density. In those iPP−SBA-15 nanocomposites, the increase of intensity took place at temperatures ranging from 95 to 120 °C and its magnitude was strongly dependent on SBA-15 content.

Change in intensity occurs now at lower temperatures, between 55 and 90 °C (as clearly depicted in Figure 10b) because the pore size in MCM-41 is much smaller than that in the SBA-15 silica: around 3.3 nm for MCM-41 and around 10 nm for SBA-15. The iPP crystals that are able to be developed within this nanometric space existing in the MCM-41 channels are, consequently, thinner than those attained in the SBA-15 pores and thus their melting takes place at inferior temperature. Intensity dependence with the MCM-41 composition is less important because the amount of iPP within the pores is much smaller in comparison with that in the SBA-15 silica. Other adverse circumstance that favors this poor pore filling is the preparation strategy since now the extrusion was used. In fact, it should be reminded that no secondary endotherm is seen in these extruded iPP−MCM-41 while a small endothermic peak was observed at same temperature interval for in situ polymerized PE−MCM-41 materials [12,13,14].

Accordingly, it could be expected that the DSC results are not able to discern the eventual endotherm arising from the confined crystallites considering the much lower amount of iPP chains inside the pores of MCM-41. Figure 6b indicates that in the case of SBA-15 that endotherm is clearly observed only for silica contents above around 8%. Fortunately, the SAXS measurements are sensitive enough to notice the existence of iPP chains within these nanometric channels in these iPP−MCM-41 composites, turning out a decisive technique for knowledge of the iPP confinement.

**Figure 11:**
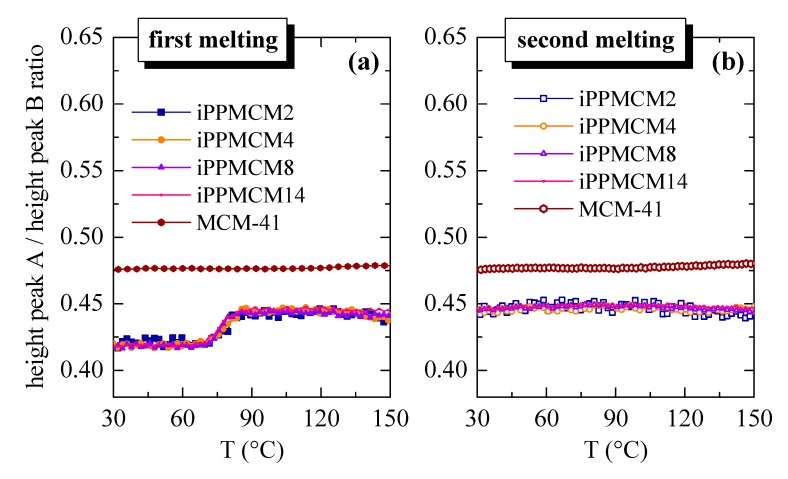
Variation with temperature of ratio between intensities of the two individual peaks, A and B (small and high intensity, respectively) that compose the MCM-41 first order, during: (**a**) first and (**b**) second melting processes.

As mentioned above, the use of synchrotron radiation allows in this commercial MCM-41 silica distinguishing a regular bimodal hexagonal arrangement with primary mesopore average diameters [54] of 3.6 and 3.3 nm for the peak with the lowest and highest intensity, respectively (these two components cannot be resolved in the case of conventional X-ray radiation). Both mesopores undergo the increase in intensity that occurs when the iPP crystallites melt to its amorphous state, as depicted in Figure 10a, but, interestingly, the ratio of intensities of the two components displays a clear jump at temperatures corresponding to the melting of confined crystallites, as observed in Figure 11a. That ratio is, however, maintained constant before and after iPP crystals main melting peak (centered at around 142 °C, as deduced from Figure 6).

Moreover, pristine MCM-41 silica does not modify the ratio between the two component peaks, since nothing changes within its pores along the whole temperature interval. It follows, therefore, that the two components with significantly different pore sizes existing in the present commercial MCM-41 silica exhibit a noticeably different behavior regarding confinement of iPP crystallites. It was described through DSC experiments [14] for in situ polymerized PE−MCM-41 that crystallinity of the “secondary” endothermic process after cooling from the molten state, i.e., along the second melting process was reduced considerably in all those specimens. That observation suggested that there was a delay in the formation of those ordered entities within MCM-41 channels in those nanocomposites because of confinement effects. Crystallites could not be developed in the same extent and size during the experimental time of the DSC test. This feature involved the diminishment of the corresponding area and the shift of that secondary peak to lower temperatures. In these iPP−MCM-41 materials, this small endotherm is not detected during DSC measurements probably because the amount of iPP within MCM-41 mesostructure is rather small. Figure 11b shows also that the ratio of the two peaks from the MCM-41 first order is maintained unchanged along the whole temperature interval during the second melting. It follows that cooling from the melt at 20 °C/min allows crystallizing the iPP chains located outside mesoporous silica, as shown in Figure 6c, but not the ones filling the MCM-41 pores. The iPP chains confined within those nanometric spaces reduce significantly their crystallization kinetics and require times longer than those involved in the SAXS experiments.

All of these results show that occurrence of confinement is characterized in these iPP−MCM-41 based materials by a substantial decrease of the melting temperatures of the polymeric chains filling the pores owing to their reduced sizes and thickness of these confined crystallites. Changes during the melting processes at the s interval of the SAXS profiles where the first order peaks for the MCM-41 are observed allow assuring the existence of iPP within these nanometric pores.

## 4. Conclusions

Nanocomposites based on isotactic polypropylene (iPP) and mesoporous silicas, either MCM-41 or SBA-15 particles, were prepared by melt extrusion. The type of silica incorporated has affected the final characteristics found in the resulting nanocomposites. Changes were, firstly, detected in the degradation behavior, with differences were dependent on experimental environment. MCM-41 particles stabilized the iPP decomposition in a less extent than SBA-15 particles under inert conditions while they contributed to increase the thermal stability of the iPP matrix under oxidative environment.

Rheological response was also influenced by the pore size of the mesoporous silica and its content. Variations in the inclusion of iPP chains within the mesostructure of these two silicas played a key role in the iPP dynamics. Beginning of a transition from a liquid to a solid-like behavior is only intuitively observed in the iPP−MCM-41 composites while rheological percolation is clearly deduced in the iPPSBA13 material.

These differences are associated with variations in the iPP confinement within these two mesoporous silicas. Nevertheless, DSC results did not provide any information on confined macrochains in the iPP−MCM-41 materials whereas a small endotherm, attributed to the melting of the confined iPP crystallites, was clearly observed in the iPP−SBA-15 composites. Real-time variable-temperature Small Angle X-ray Scattering (SAXS) experiments with synchrotron radiation were required to undoubtedly elucidate the presence of iPP macrochains within the MCM-41 pores.

SAXS profiles showed a variation with temperature during the first melting of the total area of the MCM-41 first order SAXS peak. This clear discontinuity was centered at around 70 °C. Its location appeared, then, at significantly lower temperatures than that exhibited by the composites containing SBA-15 particles since the MCM-41 pores are considerably much smaller than those in the SBA-15 silica. Accordingly, the iPP crystals that are able to be developed inside the MCM-41 pores in the iPP−MCM-41 nanocomposites are thinner than those attained in the materials incorporating SBA-15 and their melting takes place at inferior temperature. Furthermore, SAXS results on the present commercial MCM-41 silica indicated that it is actually constituted by two components with different average pore sizes, which exhibited a noticeably different behavior regarding confinement of iPP crystallites. Thus, the ratio of intensities of the two component peaks of the SAXS first order shows also a discontinuity at around 70 °C.

Precise information on the influence of MCM-41 on the iPP long spacing can be also deduced from these SAXS measurements since overlapping of the most probable iPP long spacing peak and the characteristic MCM-41 first-order diffraction does not occur in these materials because of their smaller pore size in this ordered hexagonal arrangement compared with that existing in the SBA-15 silica.

This study highlights the importance that pore size exerts in the confinement of iPP chains within the nanometric mesostructures of silicas with a subsequent effect in fundamental properties as thermal stability and dynamics of the resultant materials. They can contribute to spread out the already extensive application fields of iPP.

## Figures and Tables

**Table 1:** Characteristic decomposition temperatures (at a mass loss of 10 wt.%, T10%; and at the maximum variation, T^max^) under nitrogen or air atmosphere for neat iPP homopolymer and its composites with MCM-41 or SBA-15 particles. Estimation of silica wt.% content at a specific environment and the global average.

Sample	Inert Atmosphere			Oxidative Atmosphere				Average Silica wt.% Content
	T10% (°C)	T^max^ (°C)	Silica wt.% Content	T10% (°C)	T^max^_1_ (°C)	T^max^_2_ (°C)	Silica wt.% Content	
iPP	409	463	0	233	255	-	0	0
iPPMCM2	427	464	2.3	246	287	-	2.5	2.4
iPPMCM4	437	463	3.6	244	288	-	4.7	4.2
iPPMCM8	439	462	7.7	252	299	-	8.7	8.4
iPPMCM14	441	461	13.6	260	306	336	14.5	14.1
iPPSBA1	425	472	1.4	240	275	-	1.4	1.4
iPPSBA4	439	478	3.9	242	285	-	3.9	3.9
iPPSBA8	448	479	7.7	243	284	317	7.7	7.7
iPPSBA13	452	479	12.9	249	299	310	12.7	12.8

**Table 2:** Main melting (*T_m_*) and crystallization (*T_c_*) temperatures; overall crystallinity (normalized to the actual iPP content in the material) estimated by DSC (*f_c_*^DSC^) and WAXD (*f_c_* ^WAXD^); most probable iPP long spacing (*L*^SAXS^) determined by SAXS; and crystal size (*l_c_*) assuming a two phase model [36].

Sample	*T_m_* (°C)	*f_c_* ^DSC^	*T_C_* (°C)	*f_c_* ^WAXD^	*L*^SAXS^ (nm)	*l_c_* (nm)
iPP	142.0	0.60	114.0	0.60	10.8	6.5
iPPMCM2	142.0	0.60	112.0	0.61	10.9	6.7
iPPMCM4	142.0	0.62	112.5	0.60	11.1	6.7
iPPMCM8	141.5	0.61	112.5	0.60	10.7	6.4
iPPMCM14	142.0	0.61	112.0	0.59	10.8	6.4
iPPSBA1	143.0	0.59	111.0	0.60	10.9	6.5
iPPSBA4	143.0	0.61	111.0	0.59	11.0	6.5
iPPSBA8	142.5	0.58	110.5	0.59	11.3	6.7
iPPSBA13	142.5	0.61	110.0	0.58	11.9	6.9
